# Handbook of Anticancer Drug Development

**DOI:** 10.1038/sj.bjc.6601984

**Published:** 2004-07-27

**Authors:** B Subramanian

**Affiliations:** 1 Henry Ford Health System, Drug Discovery & Development Program, 1D-33, One Ford Place, Detroit, MI 48202, USA

I quote the editors reference in the introduction ‘A single laboratory or a small team of researchers can no longer successfully accomplish drug development. Rather there is a complex interplay among the drug discovery staff, the preclinical formulation group, toxicology and metabolism, the analytic group, large scale purification or synthesis, the clinical group and regulatory affairs.’ By quoting this, I believe, the authors have strived to achieve the best book of reference that integrates every aspect of anticancer drug development and they have succeeded in their effort. The authors have put forth four major objectives behind this book: (1) to identify potential pitfalls and pathways useful in drug development, (2) discuss methods to make preclinical and clinical process more efficient and safe, (3) let the contents of the book serve as a paradigm to rational approaches to much of drug development and (4) discuss how the complexity and nature of malignancy complicates and diverges the clinical development. All of the above objectives are well met in this book. Controversies exist in every field of research and the authors have clearly made a note of it and have included the contradicting interpretations and conclusions from individual contributors to keep the reader informed about the limitations of current knowledge.

This book consists of 33 chapters in eight independent parts covering various details of drug discovery and development through regulatory requirements for licensure. The general layout of the book is well done and the book focuses on the fundamental issues that are important to understanding and establishing a successful anticancer drug development programme. The book starts with an introduction as Part 1. Part 2 focuses on Drug Discovery, the basic and the most well done part of all covering hot topics like Proteomics, Bioinformatics and Data mining. Protein crystallography in drug discovery is the future and a dedicated chapter to it is commendable. Preclinical models, analytical techniques and delivery systems are discussed under Part 3, 4 and 5. Nanoparticle technology is emerging as a cutting-edge technology in drug development. A separate chapter on this technology would have added spice to this book. Clinical studies are briefed in Part 6. Of special interest is Part 7 that addresses certain specific issues in drug development that are not widely discussed otherwise. A quick coverage of regulatory issues is listed under Part 8. As and when needed, a simultaneous comparison of the industry practices in US and elsewhere reveals the meticulous efforts by the editors to cover as much information as possible in a more comprehensive way.

There are however certain shortcomings that probably are specific to the individual contributors. For example, Chapter 2 begins with a brief overview of the advantages of the natural environment as a rich source of natural compounds highlighting how some of the key factors like supply of material, purity, solubility, etc. result in protracted development of these compounds. Given such a lucid introduction, one would except to understand the magnitude of such factors in terms of specific examples and ways to overcome such problems to expedite the successful drug development. On the contrary, the authors have more reviewed the mechanistic aspects of specific agents grouped under three different categories, marine-, plant- and microbial-derived agents.

In summary, the book is a comprehensive representation of the overall anticancer drug developmental process and regulatory guidance essential to understanding drug development. The book is truly a ‘Handbook’ for anyone interested in or involved in anticancer drug development. Editors Daniel R Budman, Alan H Calvert and Eric K Rowinsky should be commended for their contribution to this evergrowing and challenging field of research and patient care.

